# Complete Genome Sequences of Four Isolated Bacteria from an Undergraduate Microbiology Course Using a Hybrid Assembly Approach

**DOI:** 10.1128/mra.01022-21

**Published:** 2022-02-10

**Authors:** Ciara I. Sanders, Christopher J. Ne Ville, Paul M. Orwin

**Affiliations:** a Department of Biology, California State University, San Bernardino, San Bernardino, California, USA; University of Southern California

## Abstract

Three Gram-negative bacteria and one Gram-positive bacterium were isolated from environmental samples in an undergraduate microbiology class on the basis of antibiotic resistance. Isolate DNA was purified, sequenced, and assembled using a hybrid approach. Here, we report the genomes of Acinetobacter johnsonii CSUSB1, Aeromonas hydrophila CSUSB2, Bacillus velezensis CSUSB3, and Comamonas thiooxydans CSUSB4.

## ANNOUNCEMENT

Four isolates were sequenced using the Oxford Nanopore Technologies MinION platform and the Illumina iSeq100 platform, and their genomes were assembled using a hybrid assembly approach. Initial isolations, 16S rRNA gene characterizations, and preliminary MinION sequencing were performed in an undergraduate medical microbiology course.

Acinetobacter johnsonii CSUSB1 was isolated from pond water in Highland, California. Aeromonas hydrophila CSUSB2 and Comamonas thiooxydans CSUSB4 were isolated from a water tank housing Alligator mississippiensis on the California State University, San Bernardino (CSUSB) campus. Pond and alligator water samples were plated on tryptic soy agar (TSA) plates and grown at 30°C for 7 days with five antibiotic disks. Isolates near the disks were restreaked and then retested for resistance. Colonies exhibiting resistance to the antibiotic disks (ampicillin, all isolates; erythromycin, CSUSB2 and CSUSB4; gentamicin and streptomycin, CSUSB2) were streaked for isolation on TSA plates. Single colonies were picked into 100 μL of sterile water, boiled, and centrifuged for one minute at maximum speed. The universal 16S rRNA gene primers 27f and 1492r were used to amplify the 16S rRNA gene (1) from the supernatant, and then the gene was sequenced (Retrogen Inc., San Diego, CA) using the universal 16S rRNA gene primer 530f (1). The genera of the isolates were determined using nucleotide BLAST (2), and the best hits were as follows: CSUSB1, GenBank accession number MK184297; CSUSB2, GenBank accession number NR_118547; CSUSB4, GenBank accession number NR_029161.

Genomic DNA (gDNA) for sequencing was extracted from a single colony after overnight growth in LB broth. A high-molecular-weight DNA extraction protocol (3) was used for the MinION sequencing, and the Wizard SV gDNA kit (Promega, Madison, WI) was used for the iSeq100 sequencing. Genomic libraries were prepared using the rapid barcoding library preparation kit SQK-RBk004 (Oxford Nanopore Technologies, Oxford, UK) for the MinION sequencing and the Nextera XT library preparation kit (Illumina, San Diego, CA) for the iSeq100 sequencing.

Default parameters were used for all of the following software unless otherwise specified. MinION reads (R9.4.1 flow cell) were demultiplexed with Deepbinner v 0.2.0 (4) and base called with Guppy Basecaller v 2.3.1 using the high-accuracy flip-flop algorithm (5), and adapters were removed with Porechop (Galaxy v 0.2.3) (6). FastQC (Galaxy v 0.72) and Trimmomatic (Galaxy v 0.36.5) were used to identify and clip iSeq100 reads with quality scores of <25 (7). Assembly of the long-read-only data sets using Unicycler (Galaxy v 0.4.8.0) (6) and subsequent BLAST searches led to the discovery of Acinetobacter isolate stock culture contamination with Bacillus velezensis CSUSB3. The mixed stock culture was restreaked on TSA plates, and isolated colonies were restreaked for isolation. Single colonies were picked, and gDNA was reisolated. Both isolate gDNA samples were sequenced on the iSEQ100 system, and isolated CSUSB3 gDNA was resequenced on the MinION system (R9.4.1 flow cell). The new CSUSB3 MinION reads were used as known contamination in the Unicycler (Galaxy v 0.4.8.0) long-read alignment parameters. This allowed us to use the data from the original MinION run to assemble the genome of CSUSB1.

A total of 12 circular contigs (4 chromosomes and 8 plasmids) were assembled by Unicycler (Galaxy v 0.4.8.0) (8) (Table 1). GToTree (9) was used to identify each isolate’s closest relative, and then the two-way average nucleotide identity (ANI) was used to confirm this designation (10) (Table 1).

**TABLE 1:** Genomic data

Isolate	Length (bp)	Coverage (×)	No. of Nanopore reads	Nanopore read *N*_50_ (bp)	No. of Illumina reads	GC content (%)	Contig size(s) (bp)	No. of predicted genes	GenBank assembly accession no. for best genome match (ANI [%])	GenBank accession no.
CSUSB1	3,655,004	450	17,733	4,674	373,905	41.4	3,562,289, 84,108, 8,607	3,546	GCA_004337595.1 (96.1)	CP083947 to CP083949
CSUSB2	4,923,206	272	98,634	5,384	914,196	61.4	4,846,911, 68,825, 7,470	4,538	GCA_001895965.1 (97.6)	CP083944 to CP083946
CSUSB3	4,088,756	499	30,119	5,729	634,360	46.3	4,088,756	4,041	GCA_012647845.1 (99.17)	CP083943
CSUSB4	5,642,591	318	272,391	3,217	440,231	61.4	5,441,187, 138,008, 57,673, 3,429, 2,294	5,254	GCA_000093145.2 (96.62)	CP083938 to CP083942

### Data availability.

The BioProject accession number is PRJNA767399. The GenBank accession numbers are as follows: Acinetobacter johnsonii CSUSB1, CP083947 to CP083949; Aeromonas hydrophila CSUSB2, CP083944 to CP083946; Bacillus velezensis CSUSB3, CP083943; Comamonas thiooxydans CSUSB4, CP083938 to CP083942. The BioSample accession numbers are as follows: Acinetobacter johnsonii CSUSB1 (TaxID number 40214), SAMN21540781; Aeromonas hydrophila CSUSB2 (TaxID number 644), SAMN21540782; Bacillus velezensis CSUSB3 (TaxID number 492670), SAMN21540783; Comamonas thiooxydans CSUSB4 (TaxID number 363952), SAMN21540784.
